# Quantification of PD-L1 expression and tumor mutational burden in biologically distinct advanced pancreatic cancers responding to pembrolizumab: case reports

**DOI:** 10.3389/fimmu.2024.1452543

**Published:** 2024-12-02

**Authors:** Kevin Y. Li, Andrew M. Lowy, Paul Fanta

**Affiliations:** ^1^ Department of Surgery, Division of Surgical Oncology, University of California, San Diego, San Diego, CA, United States; ^2^ Moores Cancer Center, University of California, San Diego, San Diego, CA, United States; ^3^ Division of Hematology Oncology, Department of Medicine, University of California, San Diego, San Diego, CA, United States

**Keywords:** pancreatic adenocarcinoma, immunotherapy, CPS, TMB, pancreatic neuroendocrine tumor

## Abstract

**Background:**

The advent of checkpoint therapy is one of the most important recent advancements in cancer therapy. Though checkpoint therapy is a mainstay in some cancers, it has been largely ineffective in treating cancers of the pancreas. Pancreatic ductal adenocarcinoma and pancreatic neuroendocrine tumors are seldom responsive to checkpoint inhibition.

**Case presentations:**

Here we present two cases of advanced pancreatic cancers that either failed to respond or recurred following conventional treatments. Tissue from each tumor was sequenced and analyzed for PD-L1 expression. Each patient was started on checkpoint blockade after assessing for a predictive biomarker, either the combined positive score or the tumor mutational burden. In each case, checkpoint blockade led to durable radiographic responses.

**Conclusions:**

We therefore propose that it is reasonable to assess combined positive score and tumor mutational burden in refractory or recurrent pancreatic cancers when initiation of ICB is being considered.

## Introduction

Pancreatic malignancies, such as pancreatic ductal adenocarcinoma (PDAC) and pancreatic neuroendocrine tumors (PNETs) are generally incurable when systemic disease is present, and with rare exceptions they have proven refractory to immune checkpoint blockade (ICB). The reasons for this are multiple, including a relative lack of cytotoxic T-cell (CTL) infiltration (1). Although various biomarkers for predicting ICB response are undergoing pre-clinical and clinical investigation, there is clearly no definitive test. Therefore, we are currently left to identify predictors of ICB efficacy based on existing biomarkers used in more responsive malignancies.

The Combined Positive Score (CPS) and Tumor Proportion Score (TPS) are tissue based, immunohistochemical scores that provide quantification of PD-L1 expression. Both metrics have been proposed as predictors of responsiveness to ICB in non-pancreatic malignancies. Similarly, elevated tumor mutational burden (TMB) can guide the clinical application of ICB for advanced solid tumors (2). Here, we report two cases of pancreatic malignancy with positive TPS/CPS and high TMB (TMB-H) that were associated with profound clinical responses to ICB. The first case of a patient who presented with abdominal pain, dark urine, and pruritus, and was diagnosed with pancreatic ductal adenocarcinoma. He eventually developed distant metastases which were found to have high TPS/CPS. The other case was a patient who presented with abdominal pain, and she was diagnosed with metastatic PNET. In the second case, TMB-H prompted use of immunotherapy. Both patients had significant sustained clinical responses as manifest by imaging and symptom control. We demonstrate TPS/CPS and high TMB may be predictive for response to ICB therapy in these tumor types which are generally unresponsive to ICB.

## Methods

The authors obtained informed consent from each patient to review and publish their case reports and associated imaging.

## Case presentation #1

**Figure 1:**
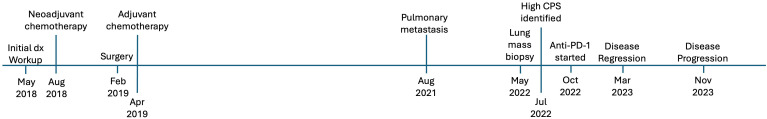
Timeline of events for case #1, a 77-year-old man who presented with pancreatic ductal adenocarcinoma.

**Figure 2:**
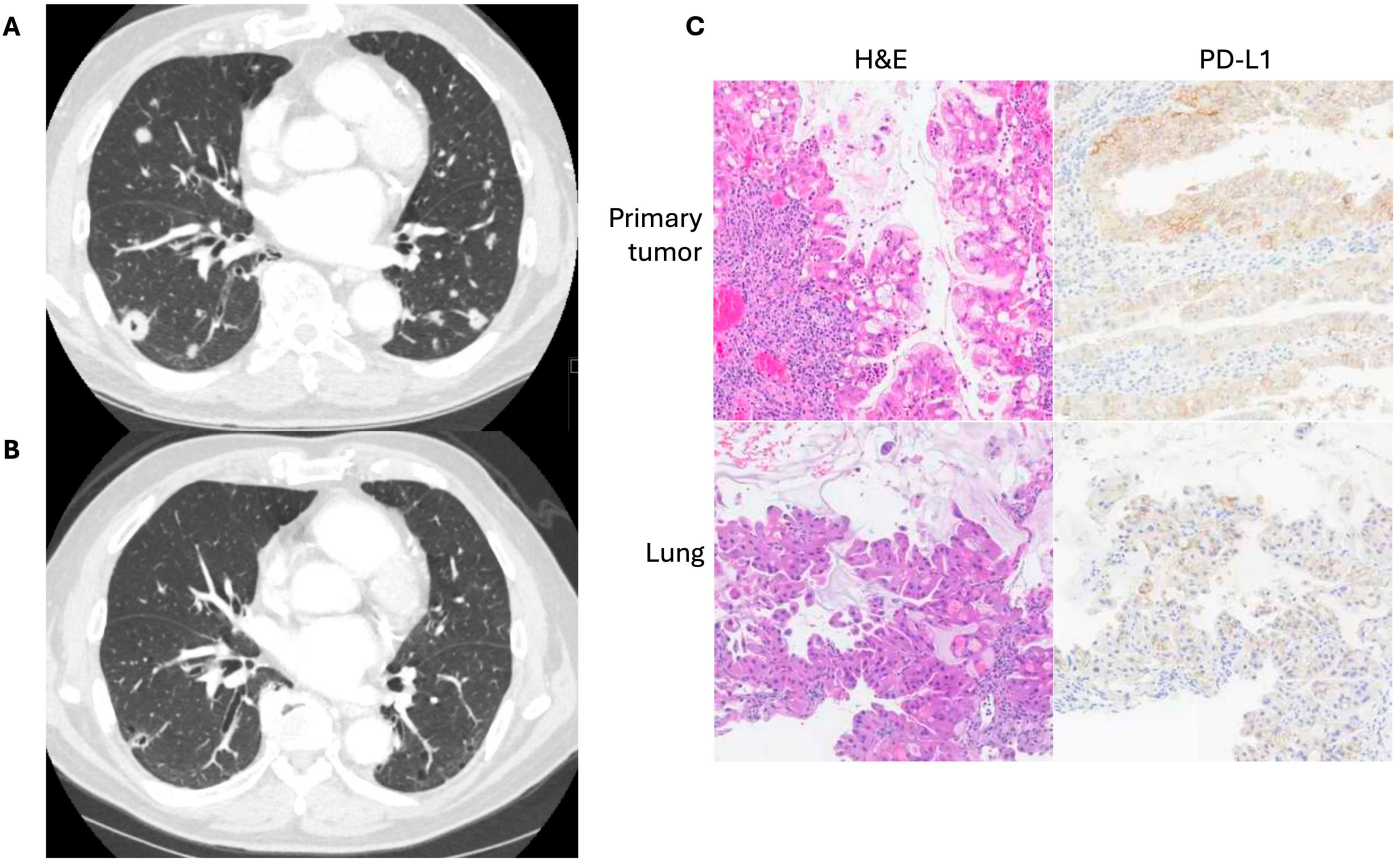
**(A)** Computed tomography imaging of pulmonary metastases prior to initiation of anti-PD-1 therapy in a patient with previously resected pancreatic ductal adenocarcinoma. **(B)** Imaging after five months of therapy. **(C)** Representative H&E and PD-L1 IHC staining of both primary tumor and lung metastases obtained from the Tempus^®^ platform.

The patient was a 77-year-old man who initially presented with abdominal pain, dark urine, and generalized pruritus (**Figure 1**). He was found to have biliary obstruction secondary to a pancreatic mass, and after endoscopic ultrasound guided biopsy, he was diagnosed with pancreatic ductal adenocarcinoma. At the time of diagnosis, he was staged as IIB (cT3N1M0). He underwent neoadjuvant chemotherapy with gemcitabine and nab-paclitaxel which was complicated by leukopenia, cellulitis, and anemia. He then underwent chemoradiation, followed by pancreaticoduodenectomy (stage ypT1aN0M0), followed by adjuvant chemotherapy with 5-fluorouracil. Over 2 years post-operatively his disease recurred with multiple pulmonary lesions concerning for metastases. He initially elected for observation. However, over time the lesions continued to grow. He ultimately pursued further treatment, and a lung lesion was biopsied. This confirmed metastatic adenocarcinoma consistent with his pancreatic primary, and he was initially started on single agent capecitabine. The biopsy sample was sent for next-generation sequencing and additional staining using the Tempus^®^ sequencing platform, which utilizes the Agilent DAKO PD-L1 (clone 22C3) antibody. PD-L1 expression on the metastatic lesion showed a CPS of 40 and TPS of 40% (**Figure 2**), and *CDH1* loss of function. In addition, retrospective PD-L1 expression testing on the primary mass revealed a CPS of 75 and a TPS of 70%, and intact mismatch repair status. Shortly after starting capecitabine, he unfortunately sustained an afferent limb perforation which necessitated exploratory laparotomy and repair. He had no post-operative complications and recovered quickly. As a result of this surgical emergency, he was taken off capecitabine. However, he was later found to have increased intrathoracic disease burden. With a positive CPS/TPS in the lung mass biopsy, he was started on pembrolizumab which was well tolerated without rash, dyspnea, or diarrhea. Follow up imaging after cycle 7 of pembrolizumab showed a marked decrease in size and number of pulmonary metastases and no metastatic disease in the abdomen or pelvis (**Figure 2**). He eventually had evidence of disease progression over one year following initiation of pembrolizumab. To date, he continues with pembrolizumab and has regular follow-up and surveillance.

## Case presentation #2

**Figure 3:**
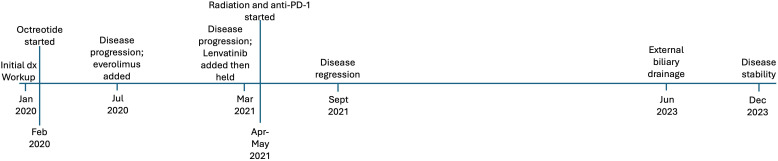
Timeline of events for case #2, a 63-year-old woman who presented with a non-functional pancreatic neuroendocrine tumor.

At the time of diagnosis, the patient was a 63-year-old female with a history of basal cell skin cancer who presented with 1 month of abdominal pain, 2 weeks of decreased appetite, and melena (**Figure 3**). Colonoscopy approximately one month prior showed diverticulosis as well as a tortuous and fixed sigmoid colon. CT scan revealed a large pancreatic mass (9 x 7cm), surrounding lymphadenopathy, and hepatic lesions. Subsequent endoscopic ultrasound showed a round, mildly hypoechoic, and moderately well-defined mass invading the main portal vein and associated portal vein thrombus. The mass also invaded the hepatic artery and the common bile duct. A common bile duct stent was placed. Fine needle aspiration revealed grade 2 neuroendocrine tumor (Ki67 15%). PET-DOTATATE scan showed a somatostatin avid mass in the central abdomen, corresponding to the pancreatic mass and periportal lymph node, but no avid hepatic lesions. She was initiated on octreotide, but experienced weight loss, a rising serum chromogranin A, and radiographic disease progression within six months of diagnosis. Repeat endoscopy showed tumor invasion of the duodenum around the biliary stent. Due to disease progression, she was started on everolimus, and octreotide was continued. Interval CT imaging showed decreased size of the pancreatic head mass (now 8.8 x 8.2cm) and decreased portal vein invasion.

**Figure 4:**
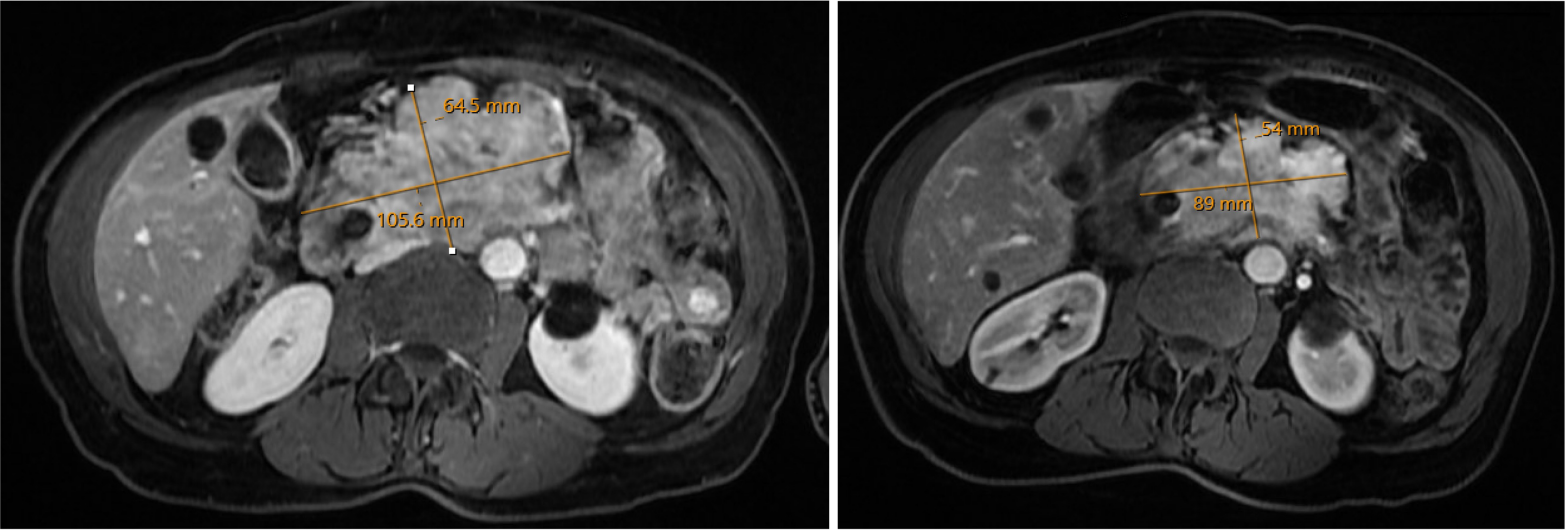
A large non-functional pancreatic neuroendocrine tumor was refractory to octreotide and everolimus. The tumor had high Tumor Mutational Burden. Imaging taken prior to initiation of anti-PD1 therapy (left) and six months after initiation (right) showed marked decrease in pancreatic tumor burden.

Molecular profiling was obtained via next-generation sequencing. This identified pathogenic alterations in TP53, TSC2, and TMB of 10.5 M/Mb, corresponding to the 89^th^ percentile. Interestingly, at that time her tumor PD-L1 expression was negative. She was referred to radiation oncology due to pain, and she completed stereotactic body radiation therapy to the primary tumor. She was maintained on octreotide and everolimus, to which pembrolizumab was added based on TMB-H status. Restaging scans 6 months after initiation pembrolizumab showed an interval decrease in the pancreatic tumor, as well as in portal hepatic lymphadenopathy (**Figure 4**). Follow up MRI at 9 months showed further improvements in tumor size. Her course was complicated by neutropenia and proctocolitis, as well as Shiga like toxin producing E coli and Clostridium difficile infection. She has continued disease control, now greater than two years since initiation of pembrolizumab, with her only additional complicating factor being biliary obstruction necessitating external drainage.

## Discussion

PDAC and PNET are typically unresponsive to ICB, with less than 5% of patients with PDAC deriving benefit from checkpoint therapy (1). In a phase I trial involving 207 patients with advanced cancer receiving anti-PD-L1 therapy, none of the 14 patients with pancreatic cancer had a response (3). In a phase II trial investigating anti-CTLA4 therapy in advanced PDAC, there were no responders (4). However, there may be a small subset of patients with pancreatic cancer who may benefit from ICB. As a result, it is important to identify additional predictors of responsiveness to checkpoint blockade in patients with PDAC, as well as PNET. In these case reports, we report patients with pancreatic malignancies who, despite an intact mismatch repair protein status, had robust, durable clinical benefit from ICB. The first case was of a patient with PDAC who presented with recurrent disease in the form of pulmonary metastasis after undergoing conventional treatments with chemotherapy and surgery. Biopsy and next-generation sequencing of a metastatic lesion showed an elevated CPS, as did the primary mass on retrospective analysis. He was therefore started on pembrolizumab as salvage therapy, and he went on to have sustained disease control for over one year. In the second case, we report a patient with locally advanced PNET who had disease progression on octreotide and everolimus. She had TMB-H status and went on to have sustained response to ICB. Taken together, these cases suggest that quantification of CPS/TPS and TMB expression may be useful to guide decisions around ICB in patients with pancreatic malignancies.

In non-pancreatic malignancies, quantification of PD-1/PD-L1 expression is commonly used as a predictor for response to ICB. In metastatic NSCLC, pembrolizumab has been established as first line monotherapy in patients without EGFR, ALK, or ROS1 mutations. In these patients, there is a positive correlation in TPS with response to pembrolizumab, with tumors having greater than 50% TPS having better response compared to tumors with 1% TPS (5, 6). CPS has also been utilized in randomized controlled trials to quantify PD-L1 expression in head and neck cancer (7), urothelial carcinoma (8), and gastric adenocarcinoma (9). The Keynote-059 trial was a single-armed multi-institutional Phase 2 trial that examined safety of pembrolizumab in patients with previously treated gastric adenocarcinoma (9). In this trial, patients with CPS ≥ 1 were enrolled and received pembrolizumab. Of 259 patients enrolled, 27% had disease control. Interestingly, only 4% of patients with available samples were found to have high microsatellite instability (MSI-H), and about half of these patients experienced objective response.

TMB is a marker that quantifies the frequency of mutational events in the genome of a tumor. It is felt to be reflective of tumor immunogenicity, and is a potential predictor for PD-L1 blockade (10). Relatively few patients with PDAC or PNET harbor high TMB (11–13). Among patients with PDAC, those with TMB-H status show signs of increased anti-tumor immune activity, and there may be a subset of patients with microsatellite stable PDAC who are TMB-H (12). In a Phase IB trial in patients with advanced neuroendocrine tumors receiving anti-PD-1 inhibition with toripalimab, those in the top 10% TMB experienced a higher objective response rate compared to those with the lowest 10% (14). Further, the Keynote-158 study prospectively investigated the association of TMB-H with responsiveness to pembrolizumab in patients with advanced solid tumors, and it included 92 patients with neuroendocrine tumors (15). Across tumor types, patients with TMB-H had longer progression-free survival compared to low TMB when treated with pembrolizumab.

Another related marker that aims to assess the degree of DNA damage in cancer is mismatch repair or microsatellite stability status. MSI-H is believed to be a predictor of solid tumor response to ICB (16). However, exceedingly few patients with PDAC are MSI-H, with only 2.4% patients found to be mismatch repair deficient in a Danish cohort, and only 0.8% in a report from Memorial Sloan Kettering Cancer Center (17). MSI-H is also rare in PNET, as shown in a prior histological review (18), though the rate of MSI-H was variable and up to 36% across different studies (19, 20). Despite its rarity, there are a subset of patients with MSS solid tumors, including pancreatic cancers, who nonetheless have a TMB-H phenotype (11–13). Taken altogether, assessing TMB may be a useful method for predicting response to ICB, and it may capture more patients than assessing for MSI would.

The tumor immune microenvironment is also a key contributor to efficacy of ICB. Among pancreatic cancers, the tumor immune microenvironment of PDAC is the most well-studied, and the presence of tumor-specific cytotoxic T-cells (CTLs) may be a prerequisite for ICB efficacy (21, 22). PDAC is characterized as an immunologically “cold” cancer, referring to the relative lack of active CTLs (1, 21, 22). Factors that contribute to this lack of tumor-specific CTLs are complex and may include the presence of myeloid cells, regulatory T-cells, the character of the tumor stroma, intercellular signaling, and the presence of specific cancer cell mutations (1, 21, 22). Myeloid cells are important to consider, as they have been implicated in tumorigenesis, immune evasion, and metastasis. To address the multiple factors that influence immune activity in pancreatic cancer, there have been several efforts to convert PDAC from a “cold” tumor to a “hot” tumor with abundant cytotoxic T-cell infiltration and activation. These strategies include upregulation of MHC-I expression by inhibiting autophagy (23), STAT3 and MEK signaling inhibition (24), and promoting anti-tumor macrophages and dendritic cells via CD40 agonism (25). In all, there are multiple variables that may influence a pancreatic tumor’s responsiveness to ICB. Though it is outside the scope of this report, it may be interesting in the future to explore the potential for quantifying cytotoxic T-cells or tumor-associated macrophages as predictors for clinical response to ICB in pancreatic cancers.

The pattern and location of disease recurrence and progression may also be important predictors of ICB efficacy in pancreatic cancers. The first case had isolated pulmonary metastases, and this may be significant and partly explain his favorable outcome. This pattern of distant recurrence is relatively uncommon, with metastasis to the liver being the most common distant site (26–28). However, isolated pulmonary recurrence may confer a more favorable prognosis. In a single institution retrospective study, 14% of PDAC recurrences were isolated pulmonary recurrences (29). This minority of patients had a significantly longer overall survival of 40.3 months, compared to just 20.9 months in patients with other patterns of recurrence (29). In a separate single institution retrospective study, patients with less than 10 isolated pulmonary metastases after resection of PDAC had a median survival of 31.3 months after disease recurrence (30). A similar survival benefit was found in a Japanese multi-center retrospective study (31), as well as in a systematic review and meta-analysis (32). In selected patients, resection of oligometastatic pulmonary recurrence may confer a survival benefit (33). In contrast, patients with isolated liver metastases may have worse outcomes, regardless of the primary tumor type. In an analysis of SEER data done by Horn et al., patients with metastatic cancer of any type with were captured and divided into two groups: those with liver metastases and those without. Patients with pancreatic cancer were included. In their multivariate analysis, the presence of liver metastasis conferred the highest hazard of death (34). In a separate retrospective study of postoperative recurrence of pancreatic cancer, patients with pulmonary recurrence had the best prognosis, while patients with hepatic recurrence had the worst prognosis in terms of overall survival (35). It is unclear why isolated pulmonary metastases are associated with better survival compared to other patterns of metastasis, and the relation to PD-1/PD-L1 blockade is understudied. Though we hypothesize that this patient’s high CPS status predicted his response to ICB, we do acknowledge that a similar patient with isolated liver metastases may not have the same outcome.

Key limitations of this report include its retrospective nature and small sample size. This is a report of two cases, and so it is not generalizable to all patients with treatment refractory pancreatic cancers. With this limited sample size, we cannot provide robust statistical analysis of treatment efficacy across multiple patients. In these cases, we are unable to determine whether the presence or absence of associated gene mutations may inhibit or complement checkpoint inhibition. In the case of the patient with PDAC, his pattern of isolated pulmonary recurrence may be a key factor in determining his favorable response to immune checkpoint blockade, and it is unclear if a similar patient with a hepatic metastasis would have the same clinical outcome. At the time of this writing, we have not identified a patient with pancreatic cancer receiving care at our institution who developed liver metastases and responded to immune checkpoint blockade. Finally, we unfortunately do not have quantification of other potentially useful biomarkers, such as CTLs or tumor myeloid cells.

To the authors’ knowledge, to date there has not been a prospective study that has examined whether CPS or TPS could predict response to ICB in pancreatic malignancies. The distribution of CPS scores in patients with a pancreatic duct adenocarcinoma has not been reported in a large series, to our knowledge, and retrospective studies have revealed that the incidence of high TMB is low. However, the cases reported here suggest that there may be a subset of patients with pancreatic cancer who have elevated CPS, TPS, or TMB and may respond to checkpoint inhibition. Therefore, these biomarkers may be useful in all cases of recurrent pancreatic cancer that are unresponsive to standard, conventional therapies.

## Data Availability

The original contributions presented in the study are included in the article/supplementary material. Further inquiries can be directed to the corresponding author.
